# Symptoms of Residential Exposure to Insecticides and Associated Factors Among Young Thai Children in Urban Areas

**DOI:** 10.3390/children11121516

**Published:** 2024-12-13

**Authors:** Pongtipat Chaiyamong, Titaporn Luangwilai, Parichat Ong-Artborirak

**Affiliations:** Department of Research and Medical Innovation, Faculty of Medicine Vajira Hospital, Navamindradhiraj University, Bangkok 10300, Thailand; p.chaiyamong2@gmail.com (P.C.); titaporn.lua@nmu.ac.th (T.L.)

**Keywords:** child, symptom, health effect, insecticide, pyrethroid, residential exposure

## Abstract

Background/Objectives: Household insecticide use may impact the health of young children in urban communities, but little is known about its acute effects. This cross-sectional study aimed to investigate the symptoms that may have been related to residential insecticide exposure and its associated factors in young children in urban areas. Methods: The study included 375 primary caregivers of children aged 6 months to 5 years from the Bangkok Metropolitan Region, Thailand, who had used insecticides in their homes within the past 6 months. An interviewer-administered questionnaire collected data on caregiver and child demographics, household insecticide use and exposure, child behaviors, and the history of child symptoms following insecticide use. Results: The findings revealed that 9.6% of young children had experienced symptoms at some point during or after household insecticide use, with coughing (66.7%), skin rash/irritation (44.4%), and runny nose (25.0%) being the most common. The final logistic regression model using backward selection indicated that factors statistically significantly associated with symptoms included being a male child (OR = 3.38; 95% CI = 1.48–7.71), hand/object-to-mouth behaviors (OR = 2.69; 95% CI = 1.26–5.74), weekly use of insecticides (OR = 2.77; 95% CI = 1.22–6.26), use of insecticide chalk (OR = 3.64; 95% CI = 1.32–10.08), and use of mosquito repellent spray/lotion (OR = 2.51; 95% CI = 1.13–5.61). Additionally, the use of insecticide spray (OR = 2.72; 95% CI = 0.97–7.65), opening doors/windows for ventilation (OR = 0.46; 95% CI = 0.21–1.02), and consistently cleaning floors with a wet cloth after use (OR = 0.52; 95% CI = 0.24–1.11) were marginally associated. Conclusions: Residential exposure to household insecticides can lead to acute health effects, primarily respiratory symptoms, in young children in urban communities. Caregivers should be informed of these health risks to reduce children’s exposure.

## 1. Introduction

Thailand’s hot and humid climate promotes the growth of disease vector insects, which in turn increases the use of insecticide products in households to control and prevent mosquitoes and other insects in order to mitigate insect-borne diseases. In densely populated urban areas like Bangkok and its vicinity, the risk of disease spread is high, leading to significant insecticide use. A national survey found that 61.6% of households in Bangkok used insecticides in the past year [1], while another study conducted in households with young children that used insecticides reported daily use at 78.8% [2].

Different types of household insecticides contain pyrethroids as key components. Exposure to pyrethroids can primarily occur through ingestion or dermal contact with contaminated dust or surface-adhering particles after domestic use [3]. Studies have revealed that floor surfaces and carpet dust are heavily contaminated with residual insecticides [4,5], and residual levels of pyrethroid insecticides have been found on the hands and feet of young children [2,6]. Additionally, the natural behaviors of young children increase the likelihood of contact with residual insecticides through ingestion, such as hand-to-mouth activities, putting fingers or objects in their mouths, and engaging in daily activities like playing, crawling, and lying on the floor, all of which elevate the risk of insecticide exposure compared to other age groups [3,7]. Residential exposure can pose health risks to young children due to their greater absorption and susceptibility to these substances [8].

Such exposure can lead to adverse outcomes—particularly neurobehavioral developmental issues—in young children. It may affect children’s short-term working memory and verbal comprehension [9], potentially resulting in behavioral disorders [10]. Additionally, exposure may increase the risk of childhood cancers, such as brain tumors and leukemia [11,12,13]. Previous studies have reported health effects of pesticide exposure related to respiratory, gastrointestinal, skin, and eye symptoms among young children living in agricultural areas [14,15]. Pesticide use at home has also been linked to children’s respiratory health [16,17,18,19]. However, little is known about the acute effects of residential exposure on children in urban communities.

Therefore, the objective of this study was to investigate the symptoms of residential exposure to insecticides following household use and the associated factors among young Thai children in urban areas. The results of this research can inform recommendations for individuals and relevant agencies to implement strategies to prevent and address issues related to residential exposure to insecticides among young children.

## 2. Materials and Methods

This research is a cross-sectional study conducted between March and April 2024 among family caregivers of young children residing in Bangkok and its surrounding areas (Thailand). Participants were selected based on the following inclusion criteria: (1) aged 18 years and older; (2) primary caregivers of children aged 6 months to 5 years who have lived in the Bangkok metropolitan area for at least 6 months; (3) a history of insecticide use in their homes within the past 6 months; (4) the ability to communicate in Thai; and (5) willingness to participate in the study. The study was promoted through community activities, public health officers, and village health volunteers at various locations, including door-to-door visits, public health service centers, community hospitals, child development centers, sub-district health-promoting hospitals, and municipalities. Participants who met the inclusion criteria were invited to participate through convenience sampling.

Sample size calculation was based on a single proportion formula [20], using a 95% confidence level, a margin of error of 0.05, and an assumed symptom prevalence of up to 40% [14,15]. The initial sample size was 369, with an additional 5% for incomplete responses, bringing the total to 388. Ultimately, 375 caregivers who provided complete data were included in the analysis. This study was approved by the Human Research Ethics Committee of the Faculty of Medicine, Vajira Hospital, Navamindradhiraj University (number COA 056-2567). Participants provided written informed consent prior to data collection.

An interviewer-administered questionnaire was used to collect data, modified from previous research [2,6,14,21,22]. It consisted of four parts: (1) caregiver characteristics, including sex, age, marital status, education level, perceived income sufficiency, relationship with their child, and duration of childcare; (2) characteristics of household insecticide use and prevention methods, including duration and frequency of insecticide use, types of insecticides (spray, coil, chalk, electric mosquito repellent), cleaning the floor with a wet cloth after use, opening doors or windows for ventilation after use, and using mosquito repellent spray/lotion; (3) child demographics and daily activities, including sex, age, chronic health conditions, sucking fingers or putting non-food objects in the mouth, frequency of hand washing, and frequency of foot washing; and (4) history of symptoms experienced following household insecticide use, validated by experts and pediatricians, including cough, skin rash/irritation, runny nose, nausea/vomiting, diarrhea, loss of appetite, eye irritation/tearing, and difficulty breathing. Participants were asked, “has a young child ever experienced any of these symptoms during or after using household insecticides (within 48 h), without having a cold or any infection?” Before data collection, the researcher explained the procedures and trained research assistants to ensure effective communication with participants and to obtain accurate data.

Data analysis was conducted using SPSS Version 28 (IBM Corp., Armonk, NY, USA). Descriptive statistics included frequency (n), percentage (%), mean, and standard deviation (SD). The chi-square test was employed to identify variables associated with symptoms of residential exposure, using a *p*-value of <0.20 for inclusion in the regression analysis. Binary logistic regression with backward (Wald) selection was performed to present odds ratios (OR) and 95% confidence intervals (95% CI) for potential factors in the final model. Notably, no multicollinearity was found in the regression model.

## 3. Results

**Figure 1:**
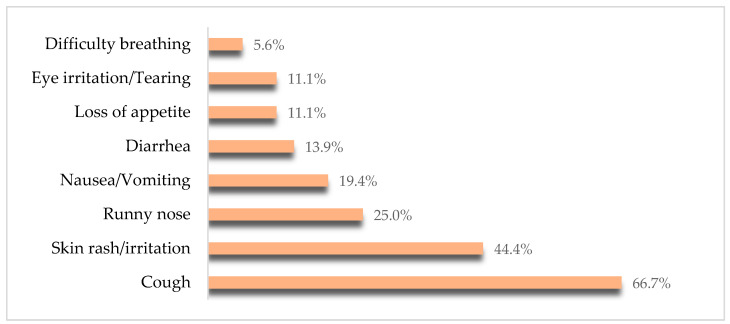
Symptoms of residential exposure to household insecticides in young children (n = 36).

Interviews with 375 caregivers revealed that 9.6% (n = 36; 95% CI = 6.6–12.6%) of young children had a history of experiencing symptoms from residential exposure to household insecticides. Only one symptom was reported by 55.5% of caregivers, two symptoms by 16.7%, and three or more symptoms by 27.8%. The most common symptoms were coughing (n = 24; 66.7%), followed by skin rash/irritation (n = 16; 44.4%), runny nose (n = 9; 25.0%), nausea and vomiting (n = 7; 19.4%), diarrhea (n = 5; 13.9%), eye irritation/tearing (n = 4; 11.1%), loss of appetite (n = 4; 11.1%), and difficulty breathing (n = 2; 5.6%) (Figure 1).

Table 1 presents the personal characteristics of young child caregivers. The mean age was 36.46 years (SD = 11.76). Most were female (81.3%), 61.6% were married, 52.0% had completed secondary or vocational education, and 52.8% perceived their income as sufficient. Additionally, 63.7% were the child’s mothers, and 37.1% had been caring for the child for 2–3 years. The chi-square test indicated that the association between caregivers’ age group and the symptoms experienced by children had a *p*-value of 0.114.

Table 2 presents the characteristics of household insecticide use, exposure, and prevention methods. The majority of households had used insecticides for 2–3 years (34.9%), with weekly usage reported at 51.8%. The most common type was spray (72.5%), followed by coil (34.4%), electric mosquito repellent (17.1%), and chalk (12.5%). The active ingredients most commonly found in these products include cypermethrin, prallethrin, imiprothrin, allethrin, phenothrin, metofluthrin, permethrin, bifenthrin, esbiothrin, and deltamethrin. About 70.7% reported always cleaning the floor with a wet cloth after use, 71.7% opened doors or windows for ventilation after use, and 48.0% used mosquito repellent spray or lotion for young children. The chi-square test showed that the association between seven factors—duration of use, frequency of use, use of spray, use of chalk, floor cleaning, ventilation, and use of mosquito repellent spray/lotion—and the symptoms experienced by children had *p*-values of 0.182, 0.023, 0.055, 0.189, 0.036, 0.137, and 0.018, respectively.

Table 3 presents the characteristics of young children. The majority were male (51.2%), with a mean age of 2.85 years (SD = 1.42). Approximately 7.7% had a chronic health condition, and 43.2% exhibited behaviors such as sucking fingers or putting non-food objects in their mouths. Handwashing was reported to occur 0–3 times per day by 41.9% of children, while foot washing at the same frequency was reported by 59.2%. The chi-square test indicated that the association between two factors—child sex and hand/object-to-mouth behaviors—and the symptoms experienced by children had *p*-values of 0.008 and 0.054, respectively.

Table 4 presents the final model of factors associated with symptoms experienced by children, derived from logistic regression using the backward (Wald) selection method. Statistically significant associations were found for being a male child (OR = 3.38; 95% CI = 1.48–7.71), hand/object-to-mouth behaviors (OR = 2.69; 95% CI = 1.26–5.74), weekly use of household insecticides (OR = 2.77; 95% CI = 1.22–6.26), use of chalk (OR = 3.64; 95% CI = 1.32–10.08), and use of mosquito repellent spray/lotion (OR = 2.51; 95% CI = 1.13–5.61). Marginal associations were noted for the use of spray (OR = 2.72; 95% CI = 0.97–7.65), opening doors or windows for ventilation after use (OR = 0.46; 95% CI = 0.21–1.02), and always cleaning floors with a wet cloth (OR = 0.52; 95% CI = 0.24–1.11).

## 4. Discussion

The findings indicated that approximately 10% of children in urban areas had ever experienced symptoms during or after household insecticide application, reflecting acute effects from residential exposure. Most symptoms were mild, including coughing, skin rashes/irritation, runny nose, and eye irritation/tearing. However, more severe symptoms were also noted, such as nausea/vomiting, diarrhea, loss of appetite, and difficulty breathing. It is possible that this study may underestimate these values by focusing on acute symptoms during and after exposure rather than considering chronic exposure or chronic symptoms. Unlike in adults, symptoms reported by caregivers for children may not adequately include nervous system issues, as these symptoms may not be as clearly evident. A previous study of children in Bangkok showed a negative association between exposure to pyrethroids and GABA concentration, a neurotransmitter [22]. Although young children may primarily be exposed to insecticides through ingestion or dermal contact [3], they can also resuspend dust during play, and their lower breathing zone, being close to the floor, may result in higher inhalation exposure [23]. They are also exposed to pyrethroids through their diets, though this is less than from residential sources [24]. These findings suggest that young children in urban areas are environmentally exposed to insecticides via multiple pathways, with residential exposure being a primary source, potentially increasing the risk of adverse health effects. This highlights the need for caregivers to be informed about health risks related to residential exposure and the routes of exposure to contaminated residues [21].

A cross-sectional study of Lebanese children found that exposure to pesticides from domestic use was significantly associated with respiratory diseases (OR = 1.77), asthma (OR = 1.99), and chronic respiratory symptoms, including chronic phlegm (OR = 1.96), chronic wheezing (OR = 1.49), and ever wheezing (OR = 1.50) [16]. Similarly, pesticide use at home was significantly associated with the presence of chronic wheezing (OR = 1.91) in Lebanese children [17]. In Chinese children, exposure to residential insecticides increased the risk of respiratory diseases, including recurrent infections [18]. Furthermore, pesticide use in the kitchen or dining rooms was significantly associated with increased odds of wheezing and dry cough in U.S. children [19]. In studies conducted in agricultural areas, pesticide exposure has been shown to affect respiratory and allergic outcomes in 5-year-old children, with coughing as the most frequent symptom (39%), and pyrethroid exposure has also been associated with wheezing and itchy rashes [15]. Another study on pesticide exposure in young Thai children found that caregivers reported symptoms during the dry and rainy seasons, with the most common symptoms related to the respiratory, gastrointestinal, and integumentary systems [14]. Variations in the proportions of symptoms may result from differences in study areas, population characteristics, seasonal factors, time periods, and, most notably, exposure characteristics such as application methods, intensity levels, and the types of active ingredients in products. Even though nearly all household insecticide products examined in this study contain pyrethroid components, which are considered less toxic, the findings suggest that their use may have acute effects on urban children, primarily impacting their respiratory health.

In addition, the study found that child sex and hand/object-to-mouth behaviors were associated with symptoms experienced by children. This may be due to gender influencing the types of behaviors and activities in which children engage, resulting in a greater chance of exposure [25]. It may also affect the toxicokinetics of pyrethroid metabolites [26], with the pyrethroid type potentially influencing differences in toxicity between the sexes [27]. Sucking fingers or putting non-food objects in the mouth are natural behaviors in young children that may increase their risk of exposure to residual insecticides when their hands and toys come into contact with contaminated surfaces or dust in the home [2,4,5,6]. A previous study has shown that object-to-mouth behaviors are associated with exposure to pyrethroids, as assessed by metabolite concentrations in urine [22]. The findings suggest that certain child behaviors may increase the risk of residential insecticide exposure via the oral route.

The frequency of household insecticide use, the type of insecticide, and the use of mosquito repellent spray/lotion were potential factors associated with symptoms experienced by children. This may be due to the fact that increased frequency of use can heighten the chance of exposure, potentially leading to these symptoms. Similarly, the frequency of insecticide use was positively associated with home dust concentrations of pyrethroid insecticides [28] and with pyrethroid metabolites in urine [22]. A qualitative study revealed that mothers and fathers of children were concerned about respiratory tract irritation with the use of insecticide sprays [29]. Using insecticide sprays can transfer residues to food, resulting in excessive dietary exposure for children [30]. Spray applications in areas of the home where children spend significant time may lead to increased inhalation and dermal exposure [31]. While pediatric exposure to insecticide chalk can occur via multiple routes—including ingestion, dermal contact, and inhalation—it may lead to clinical effects such as vomiting, nausea, dermal irritation, diarrhea, and coughing [32]. A study in Thailand found that the use of sprays and chalk was positively correlated with the concentration of pyrethroids on the hands and feet of children, thereby increasing the risk of exposure [6]. Furthermore, children may experience reactions, such as rashes, from insect repellents [33]. While several DEET-based insect repellents are approved as safe and effective when used correctly, it is advisable to take precautions when applying them to children [34].

Floor cleaning with a wet cloth and ventilation after use were protective factors marginally associated with symptoms experienced by children. Opening doors or windows may help dilute the concentration of insecticides, while floor cleaning reduces contamination from residues on surfaces, thereby decreasing the risk of exposure via multiple routes and lowering symptoms in children. Previous studies support this, finding that ventilation and cleaning immediately after application may reduce insecticide exposure for residents [31,35]. Although decontaminating rooms has little effect on pyrethroid concentration in suspended particles, it significantly reduces contamination levels in house dust and on furniture surfaces [35]. Similarly, previous research in Thailand found that floor cleaning was marginally associated with reduced pyrethroid exposure levels in young children (OR = 0.43, *p*-value = 0.079) [22].

This study has several limitations, including a cross-sectional design that restricts causal inference and non-probability sampling, which may not fully represent the population, affecting generalizability. Information and recall bias may arise as caregivers report acute symptoms based on their perceptions. Due to the limited information in the questionnaire used in this study, further research is needed to explore the prevalence of symptoms, including data on frequency, severity, and duration, as well as definitive diagnoses. Future studies should collect environmental and biological samples to assess insecticide exposure and investigate chronic symptoms in young children. Additionally, immigrant populations should be included, and a qualitative approach should be considered. Nonetheless, this preliminary research highlights acute symptoms associated with residential insecticide exposure in children, which is a significant source of concern regarding adverse health effects.

## 5. Conclusions

Using household insecticides can lead to low levels of residential exposure and may potentially result in acute symptoms, particularly affecting the respiratory system, in young Thai children in urban settings. Several factors are associated with these symptoms, including child characteristics, the frequency and type of insecticides used, and appropriate practices following application. Proper ventilation and floor cleaning can possibly reduce children’s exposure and the risk of adverse symptoms. Caregivers and parents should minimize exposure to prevent both acute and chronic effects. Therefore, actions should be taken to raise awareness of the hazards of household insecticides and promote their proper use in mitigating exposure and health risks to children, such as by providing educational interventions or activities through public healthcare services.

## Figures and Tables

**Table 1:** Personal characteristics of young child caregivers classified by children’s symptoms from residential insecticide exposure (n = 375).

Personal Characteristics of Young Child Caregivers	Total	Symptoms [n (%)]		*p*-Value ^†^
	n (%)	No	Yes	
Sex				0.439
Male	70 (18.7)	65 (92.9)	5 (7.1)	
Female	305 (81.3)	274 (89.8)	31 (10.2)	
Age				0.114
<30 years	116 (30.9)	108 (93.1)	8 (6.9)	
30–39 years	137 (36.5)	126 (92.0)	11 (8.0)	
40–49 years	70 (18.7)	58 (82.9)	12 (17.1)	
≥50 years	52 (13.9)	47 (90.4)	5 (9.6)	
Marital Status				0.486
Single	101 (26.9)	90 (89.1)	11 (10.9)	
Married	231 (61.6)	208 (90.0)	23 (10.0)	
Divorced/Widowed/Separated	43 (11.5)	41 (95.3)	2 (4.7)	
Education level				0.649
Primary school or lower	49 (13.1)	46 (93.9)	3 (6.1)	
Secondary school/Vocational	195 (52.0)	176 (90.3)	19 (9.7)	
Higher vocational and above	131 (34.9)	117 (89.3)	14 (10.7)	
Perceived income sufficiency				0.723
Insufficient	177 (47.2)	159 (89.8)	18 (10.2)	
Sufficient	198 (52.8)	180 (90.9)	18 (9.1)	
Relationship with child				0.516
Father	61 (16.3)	57 (93.4)	4 (6.6)	
Mother	239 (63.7)	213 (89.1)	26 (10.9)	
Others	75 (20.0)	69 (92.0)	6 (8.0)	
Duration of childcare				0.437
6 months–1 year	115 (30.7)	105 (91.3)	10 (8.7)	
2–3 years	139 (37.1)	128 (92.1)	11 (7.9)	
>3 years	121 (32.3)	106 (87.6)	15 (12.4)	

^†^ Chi-square test.

**Table 2:** Characteristics of household insecticide use and prevention methods classified by children’s symptoms from residential insecticide exposure (n = 375).

Characteristics of Household Insecticide Use	Total	Symptoms [n (%)]		*p*-Value ^†^
	n (%)	No	Yes	
Duration of household insecticide use				0.182
6 months–1 year	128 (34.1)	118 (92.2)	10 (7.8)	
2–3 years	131 (34.9)	121 (92.4)	10 (7.6)	
>3 years	116 (30.9)	100 (86.2)	16 (13.8)	
Frequency of household insecticide use				0.023
Weekly	193 (51.5)	168 (87.0)	25 (13.0)	
Monthly	182 (48.5)	171 (94.0)	11 (6.0)	
Using spray				0.055
No	103 (27.5)	98 (95.1)	5 (4.9)	
Yes	272 (72.5)	241 (88.6)	31 (11.4)	
Using coil				0.820
No	246 (65.6)	223 (90.7)	23 (9.3)	
Yes	129 (34.4)	116 (89.9)	13 (10.1)	
Using chalk				0.189
No	328 (87.5)	299 (91.2)	29 (8.8)	
Yes	47 (12.5)	40 (85.1)	7 (14.9)	
Using electric mosquito repellent				0.947
No	311 (82.9)	281 (90.4)	30 (9.6)	
Yes	64 (17.1)	58 (90.6)	6 (9.4)	
Cleaning the floor with a wet cloth				0.036
Sometimes	110 (29.3)	94 (85.5)	16 (14.5)	
Always	265 (70.7)	245 (92.5)	20 (7.5)	
Ventilation after use				0.137
No	106 (28.3)	92 (86.8)	14 (13.2)	
Yes	269 (71.7)	247 (91.8)	22 (8.2)	
Using mosquito repellent spray/lotion				0.018
No	195 (52.0)	183 (93.8)	12 (6.2)	
Yes	180 (48.0)	156 (86.7)	24 (13.3)	

^†^ Chi-square test.

**Table 3:** Characteristics of household insecticide use and prevention methods classified by children’s symptoms from residential insecticide exposure (n = 375).

Characteristics and Daily Activities of Young Children	Total	Symptoms [n (%)]		*p*-Value ^†^
	n (%)	No	Yes	
Sex				0.008
Male	192 (51.2)	166 (86.5)	26 (13.5)	
Female	183 (48.8)	173 (94.5)	10 (5.5)	
Age				0.673
<1 year	43 (11.5)	38 (88.4)	5 (11.6)	
1 year	69 (18.4)	65 (94.2)	4 (5.8)	
2 years	86 (22.9)	80 (93.0)	6 (7.0)	
3 years	69 (18.4)	61 (88.4)	8 (11.6)	
4 years	70 (18.7)	62 (88.6)	8 (11.4)	
5 years	38 (10.1)	33 (86.8)	5 (13.2)	
Chronic health condition				0.505
No	346 (92.3)	314 (90.8)	32 (9.2)	
Yes	29 (7.7)	25 (86.2)	4 (13.8)	
Hand/Object-to-mouth behaviors				0.054
No	213 (56.8)	198 (93.0)	15 (7.0)	
Yes	162 (43.2)	141 (87.0)	21 (13.0)	
Frequency of hand washing				0.865
0–3 times per day	157 (41.9)	143 (91.1)	14 (8.9)	
4–6 times per day	151 (40.3)	135 (89.4)	16 (10.6)	
>6 times per day	67 (17.9)	61 (91.0)	6 (9.0)	
Frequency of foot washing				0.911
0–3 times per day	222 (59.2)	201 (90.5)	21 (9.5)	
>3 times per day	153 (40.8)	138 (90.2)	15 (9.8)	

^†^ Chi-square test.

**Table 4:** Final model of factors associated with symptoms from residential insecticide exposure in young children using backward binary logistic regression.

Variable	B	SE	*p*-Value	OR	95% CI
Constant	−3.67	1.76	0.037	0.03	
Sex of child (male)	1.22	0.42	0.004	3.38	1.48, 7.71
Hand/Object-to-mouth behaviors (yes)	0.99	0.39	0.010	2.69	1.26, 5.74
Frequency of household insecticide use (weekly)	1.02	0.42	0.014	2.77	1.22, 6.26
Using spray (yes)	1.00	0.53	0.057	2.72	0.97, 7.65
Using chalk (yes)	1.29	0.52	0.013	3.64	1.32, 10.08
Using mosquito repellent spray/lotion (yes)	0.92	0.41	0.024	2.51	1.13, 5.61
Ventilation after use (yes)	−0.77	0.40	0.055	0.46	0.21, 1.02
Cleaning the floor with a wet cloth (always)	−0.66	0.39	0.091	0.52	0.24, 1.11

Note: B, unstandardized coefficients; SE, standard error; OR, odds ratio; 95% CI, 95% confidence interval for OR.

## Data Availability

The data are available upon request from the corresponding author. The data are not publicly available due to ethical reasons.
